# Health Workforce Brain Drain: From Denouncing the Challenge to Solving the Problem

**DOI:** 10.1371/journal.pmed.1001514

**Published:** 2013-09-17

**Authors:** Giorgio Cometto, Kate Tulenko, Adamson S. Muula, Ruediger Krech

**Affiliations:** 1 Global Health Workforce Alliance, World Health Organization, Geneva, Switzerland; 2 IntraHealth International, Washington (D.C.), United States of America; 3 College of Medicine, University of Malawi, Blantyre, Malawi; 4 Department of Ethics and Social Determinants of Health, World Health Organization, Geneva, Switzerland

## Abstract

Giorgio Cometto and colleagues discuss the study by Tankwanchi and colleagues on physician migration to the United States from sub-Saharan Africa and the steps that the US, other destination countries, and SSA countries can take to address the problem.

*Please see later in the article for the Editors' Summary*

## The Challenge of Health Workforce Brain Drain

Migration of health workers from low- and middle-income countries (LMICs) to high-income countries is one of the most controversial aspects of globalization, having attracted considerable attention in the health policy discourse at both the technical and political level [1]. Some countries (e.g., the Philippines [2]) train health workers to export them overseas and reap the financial benefits of remittances; such investments should therefore be considered as driven primarily by economic—rather than population health—motives. In most cases, however, migration of health professionals is unplanned for and represents a “brain drain” for source countries, a result of enormous wage differences and poor working conditions, including lack of support, adequate infrastructure, and career development opportunities, in LMICs. The increased recognition that this phenomenon contributes to exacerbating human resources for health (HRH) shortages in LMICs culminated in the adoption of the World Health Organization global code of practice on international recruitment of health personnel (the WHO Code) at the World Health Assembly in 2010 [3].

Four countries (US, UK, Australia, Canada) together employ 72% of foreign-born nurses and 69% of doctors working in the Organisation for Economic Co-operation and Development 

(OECD) bloc [4], with the US employing the most of any country. In this week's issue of *PLOS Medicine*, a new analysis of physician migration from sub-Saharan Africa (SSA) to the United States by Tankwanchi and colleagues [5] highlights two inter-related and worrying trends: (1) migration of these physicians is on the rise; and (2) physician density is declining in the majority of African countries under investigation.

The WHO Code represents an unprecedented opportunity for countries to collaborate on tackling the negative effects of HRH migration. In addition to the migration-specific measures (such as promoting bilateral or multilateral agreements to coordinate HRH flows between countries, or advocating fair treatment of migrant health workers), the Code recognizes the interconnectedness of HRH actions in the national and global health labor markets, and the need for a systemic approach to health workforce development, recommending that countries should aim towards self-sufficiency in HRH production, and put in place the regulation, education, and management policies, and incentives to enhance retention.

## Understanding the Root Causes of Migration

Adopting effective policies to address international health workforce migration requires both understanding the local drivers of (inward or outward) migration, as well as identifying evidence-based policy options. In the case of the US, challenges include chronic under-investment in physician training and a failure to select trainees from under-served areas; additionally, licensing creep for some fields translates into higher training costs and educational debt loads, resulting in higher wage demands and a reluctance by US-trained health workers to serve in low-income areas [6]–[10]. The paucity of US-trained physicians willing to serve in rural areas is balanced by doctors from overseas willing to accept such postings, although after their initial service is complete, foreign-trained doctors have been shown to be more likely to practice in major metropolitan areas than US-trained doctors [11]. Migration of physicians from SSA to the US therefore may be happening more among doctors from privileged communities who feel they do not fit in rural SSA where they are needed, but who can accept—at least on a temporary basis—the rural North American environment [12].

Recurrent problems that affect most SSA countries include under-investment in HRH education, inadequate attention to supportive management practices, limited opportunities for career progression and professional development, and poor remuneration and incentives [13]. These challenges are often the result of misconceived macro-economic policies, as wage ceilings prevent meeting needs for health personnel [14].

## The Need for Evidence-Based Solutions

Of course, denouncing the negative effects of international HRH migration and identifying its root causes are not enough; shifting to a problem-solving mode requires identifying and putting in place effective solutions. With regard to destination countries, experience from the UK shows that ethical guidance alone was ineffective in preventing inflow of doctors between 2001 and 2004, due to competing priorities of the NHS. Only when the UK increased domestic training numbers, changed immigration laws, and introduced bilateral agreements with source countries did new registrations of overseas physicians decline drastically (from 3,206 in 2003 to 4 in 2004 from South Africa; from 4,626 in 2004 to 1,169 in 2007 from Pakistan) [15].

Some source countries are also taking action: facing a dramatic shortfall of health workers in the early 2000s, Malawi implemented an Emergency Human Resources Programme (EHRP), which boosted domestic training of health workers and facilitated in-country retention through a variety of measures, including a 52% salary top-up for priority cadres, and an expansion of opportunities for post-graduate training. Between 2004 and 2009, the EHRP increased the number of health care workers by 53%, raising the HRH density from 0.87 to 1.44/1,000 population [16]. However, these gains are vulnerable, as donors are now shifting their support elsewhere. India is considering requiring physicians to certify that they will return home before they may receive a visa to train and practice in the US [17].

Simply increasing the wages of physicians in LMICs is not effective, as many more factors such as working conditions, housing, and career advancement play a role in their departure. The current nurse and midwife strike in Liberia [18], motivated in large part by wage demands, exemplifies the challenges of retaining physicians through selective salary increments when these are not matched by corresponding improvements for other health workers.

## Policy Options for Low- And Middle-Income Countries

For most LMICs, matching the levels of remuneration and the opportunities for specialized training and research available in advanced economies is not realistic in the short-term, nor is it desirable given the competing needs for use of limited public resources. A recent literature review has identified realistic strategies of proven effectiveness to limit the negative effects of HRH migration on LMIC [19], including:

- placing greater emphasis on non-wage retention strategies, including improving working and living conditions [20];
- diversifying the skills mix to harness the potential of non-physician clinicians [21] and community health workers [22]; the credentials awarded to these cadres are typically recognized only in their own country, making them less vulnerable to international migration;
- circular migration, i.e., promoting a triangular flow of talent and skills by encouraging some migrant health workers to return to their home country [23].

## What High-Income Countries Can Do

In parallel, OECD countries could decrease their reliance on foreign-trained health workers by:

- increasing investment in domestic health professional education and aligning government educational spending with employment opportunities. For example, an analysis showed that the US had 116,000 nursing vacancies [24], while new college graduates in other fields face unemployment rates of 53% [25];
- piloting or expanding innovative financing mechanisms, for instance allowing local and private entities to provide complementary funding to the (US) federal government's National Health Service Corps, a bonding system whereby physicians receive scholarships or educational loan repayment in exchange for guaranteed service in underserved communities;
- not hiring from the countries with the worst health care worker-to-population ratios;
- adopting a shared regulation approach (i.e., with inputs from government, patient groups, and major health care payers) to health professional schools and health professions, which are now entirely self-regulated;
- encouraging the development of more cost-effective ways to educate health professionals through innovation grants;
- planning a more diversified skill mix for the health team, better harnessing the complementarity of different cadres, including nurse practitioners, physician assistants, medical assistants, and primary care emergency medical technicians [26].

## Commitments to Change

The upcoming Third Global Forum on Human Resources for Health, to be held in Recife, Brazil, on 10–13 November 2013, will provide an opportunity to commit to the policy and investment decisions that need to be adopted for a sustainable and systemic strengthening of the health workforce, as a critical element in the drive towards universal health coverage [27]. Certain aspects of health workforce development are the exclusive prerogative of national governments, and only political commitment at the national level can trigger the transformative action required in health workforce development efforts. Other aspects, prominent among which is the issue of international migration, require global solidarity and coordination. The WHO Code provides a valid framework for such collaborative efforts and for monitoring their effectiveness. The sobering findings of the analysis by Tankwanchi and colleagues highlight how much remains to be done to accelerate its implementation.
